# Network medicine‐based analysis of the hepatoprotective effects of *Amomum villosum* Lour. on alcoholic liver disease in rats

**DOI:** 10.1002/fsn3.4046

**Published:** 2024-02-22

**Authors:** Jing Wei, Sihua Wang, Junze Huang, Xinhua Zhou, Zhengming Qian, Tingbiao Wu, Qing Fan, Yongyin Liang, Guozhen Cui

**Affiliations:** ^1^ School of Bioengineering Zhuhai Campus of Zunyi Medical University Zhuhai China; ^2^ Guangzhou Eighth People's Hospital Guangzhou Medical University Guangzhou China; ^3^ Dongguan HEC Cordyceps R&D Co., Ltd. Dongguan China; ^4^ Basic Medical Science Department Zhuhai Campus of Zunyi Medical University Zhuhai China

**Keywords:** alcoholic liver disease, *Amomum villosum  Lour.*, hepatoprotective effects, network medicine

## Abstract

Alcoholic liver disease (ALD) is characterized by high morbidity and mortality, and mainly results from prolonged and excessive alcohol use. *Amomum villosum* Lour. (*A*. *villosum*), a well‐known traditional Chinese medicine (TCM), has hepatoprotective properties. However, its ability to combat alcohol‐induced liver injury has not been fully explored. The objective of this study was to investigate the hepatoprotective effects of *A*. *villosum* in a rat model of alcohol‐induced liver disease, thereby establishing a scientific foundation for the potential preventive use of *A*. *villosum* in ALD. We established a Chinese liquor (Baijiu)‐induced liver injury model in rats. Hematoxylin and eosin (HE) staining, in combination with biochemical tests, was used to evaluate the protective effects of *A*. *villosum* on the liver. The integration of network medicine analysis with experimental validation was used to explore the hepatoprotective effects and potential mechanisms of *A*. *villosum* in rats. Our findings showed that *A*. *villosum* ameliorated alcohol‐induced changes in body weight, liver index, hepatic steatosis, inflammation, blood lipid metabolism, and liver function in rats. Network proximity analysis was employed to identify 18 potentially active ingredients of *A*. *villosum* for ALD treatment. These potentially active ingredients in the blood were further identified using mass spectrometry (MS). Our results showed that *A*. *villosum* plays a hepatoprotective role by modulating the protein levels of estrogen receptor 1 (ESR1), anti‐nuclear receptor subfamily 3 group C member 1 (NR3C1), interleukin 6 (IL‐6), and tumor necrosis factor‐α (TNF‐α). In conclusion, the results of the current study suggested that *A*. *villosum* potentially exerts hepatoprotective effects on ALD in rats, possibly through regulating the protein levels of ESR1, NR3C1, IL‐6, and TNF‐α.

## Graphical abstract

This study investigates the hepatoprotective properties of *Amomum villosum* Lour. (*A*. *villosum*) in a rat model of alcohol‐induced liver disease (ALD), induced by Chinese liquor (Baijiu) consumption. *A*. *villosum* demonstrated significant protective effects against alcohol‐induced changes in body weight, liver index, hepatic steatosis, inflammation, blood lipid metabolism, and liver function. Network medicine analysis identified potentially active ingredients, and experimental validation revealed that *A*. *villosum* may exert hepatoprotective effects by modulating the protein levels of estrogen receptor 1 (ESR1), anti‐nuclear receptor subfamily 3 group C member 1 (NR3C1), interleukin 6 (IL‐6), and tumor necrosis factor‐α (TNF‐α).

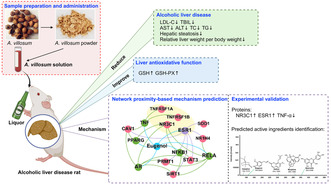

## 1 INTRODUCTION

Alcohol consumption constitutes a substantial global health concern, leading to far‐reaching consequences, including an estimated 3 million fatalities and over 130 million disability‐adjusted life years (DALYs) worldwide in 2016 (Shield et al., 2020). This problem is particularly pronounced in China, where alcohol is associated with more than 200 distinct diseases, predominantly in men (Im et al., 2023). The issue appears to be worsening, as global per‐capita alcohol consumption rose from 1990 to 2017, with projections indicating continued growth through 2030 (Manthey et al., 2019). At the forefront of the health challenges posed by alcohol is alcoholic liver disease (ALD), a complex spectrum of liver disorders. Chronic alcohol consumption can lead to ALD through a series of progressive hepatic pathologies that include steatosis, steatohepatitis, and cirrhosis. These conditions are primarily induced by alcohol‐driven inflammation, lipid accumulation, and oxidative stress (Aslam & Kwo, 2023; Ponziani et al., 2015). As a central orchestrator of alcohol metabolism, the liver processes a substantial portion of ingested alcohol. This metabolic pathway involves the transformation of alcohol into acetaldehyde, a process facilitated by enzymes such as alcohol dehydrogenase and the microsomal ethanol oxidizing system (Hyun et al., 2021). Chronic and excessive alcohol consumption can result in elevated hepatic acetaldehyde contents, evoking hepatotoxic effects, including liver cell necrosis, inflammation, and fibrosis, thereby instigating ALD (Neuman et al., 2022; Teschke, 2018). The management of liver damage induced by alcohol consumption remains a vital area of research and clinical focus. Notably, silybin, a medicinal derivative of milk thistle, has demonstrated therapeutic potential for treating acute and chronic liver injuries. However, its oral administration may result in gastrointestinal side effects, limiting its use (Loguercio & Festi, 2011). Therefore, the development of safe and effective hepatoprotective agents continues to be an essential and urgent task in addressing this health issue.

*Amomum villosum* Lour. (*A*. *villosum*), also known as “Sha Ren” in traditional Chinese medicine (TCM), is a plant in the Zingiberaceae family. The name of the plant was further checked in World Flora Online (www.worldfloraonline.org), accessed on August 08, 2023. This tropical plant is a medicinal food homology and mainly flourishes in Southeast Asia and South China. Current pharmacological studies indicate that its main constituents include volatile oils, flavonoids, carbohydrates, organic acids, and inorganic compounds, which collectively contribute to a broad spectrum of biological activities (Yong et al., 2020). Notably, the hepatoprotective attributes of *A*. *villosum* are particularly noteworthy. It has potential as a nutritional supplement in the management of liver conditions. For example, previous studies have shown that *A*. *villosum* mitigates carbon tetrachloride (CCl_4_)‐induced liver injury in mice, displaying notable antioxidant and hepatoprotective activities (Meng et al., 2020). Furthermore, the volatile oil of *A*. *villosum* has been demonstrated to inhibit nonalcoholic fatty liver disease (NAFLD) through mechanisms involving the gut–liver axis (Lu et al., 2018). Similarly, the ethyl acetate fraction of *A*. *villosum* var. xanthioides has been found to alleviate hepatic endoplasmic reticulum stress‐induced nonalcoholic steatohepatitis (NASH), likely through the enhancement of antioxidant capacities (Cho et al., 2021). These documented hepatoprotective properties of *A*. *villosum* underline its therapeutic potential, stimulating current scientific interest. Nonetheless, its effects on alcohol‐induced liver injury remain largely unexplored and thus call for more comprehensive studies.

Network pharmacology, which intersects systems biology, computer information technology, and pharmacology, has become increasingly pivotal in elucidating the pharmacological mechanisms of traditional medicines. For instance, studies have utilized network pharmacology to decipher the intricate mechanisms underlying the therapeutic efficacy of various herbs, revealing insights into their pathways (L. Li et al., 2023). This approach has demonstrated its effectiveness in various contexts, including the treatment of chronic diseases such as gastrointestinal and neurological diseases (Sharma et al., 2022, 2023; Thakur et al., 2023), demonstrating its versatility and depth in traditional medicine research. Despite these advancements, the field of network pharmacology is undergoing significant progress, transitioning toward the more encompassing and sophisticated framework of network medicine. This transition marks a critical expansion in scope, from a focus on individual pharmacological agents to an integrated understanding of disease mechanisms, therapeutic targets, and drug interactions at a system‐wide level.

Network medicine has emerged as a groundbreaking discipline poised to transform our comprehension of human diseases and therapeutic interventions. This field leverages cutting‐edge methods to elucidate the complex relationships between genes associated with specific diseases and prospective therapeutic targets (Barabási et al., 2011). Pioneering this approach, Guney et al. developed an innovative, unsupervised, and unbiased framework within network medicine. Their approach illuminates the multifaceted interactions between drugs and diseases by synthesizing data from protein–protein interactions, and associations between drugs, diseases, and drug targets. To mitigate the inherent biases in human interactomes, they proposed a novel drug–disease proximity measurement method. This methodology quantifies the distances between clusters of drug targets and disease‐related proteins, offering insights into the potential therapeutic efficacy of drugs for specific diseases (Guney et al., 2016). Furthermore, this approach facilitates in‐depth exploration of the interactions between drug targets and disease proteins, enhancing our understanding of drug efficacy and mechanism (Guney et al., 2016).

**FIGURE 1:**
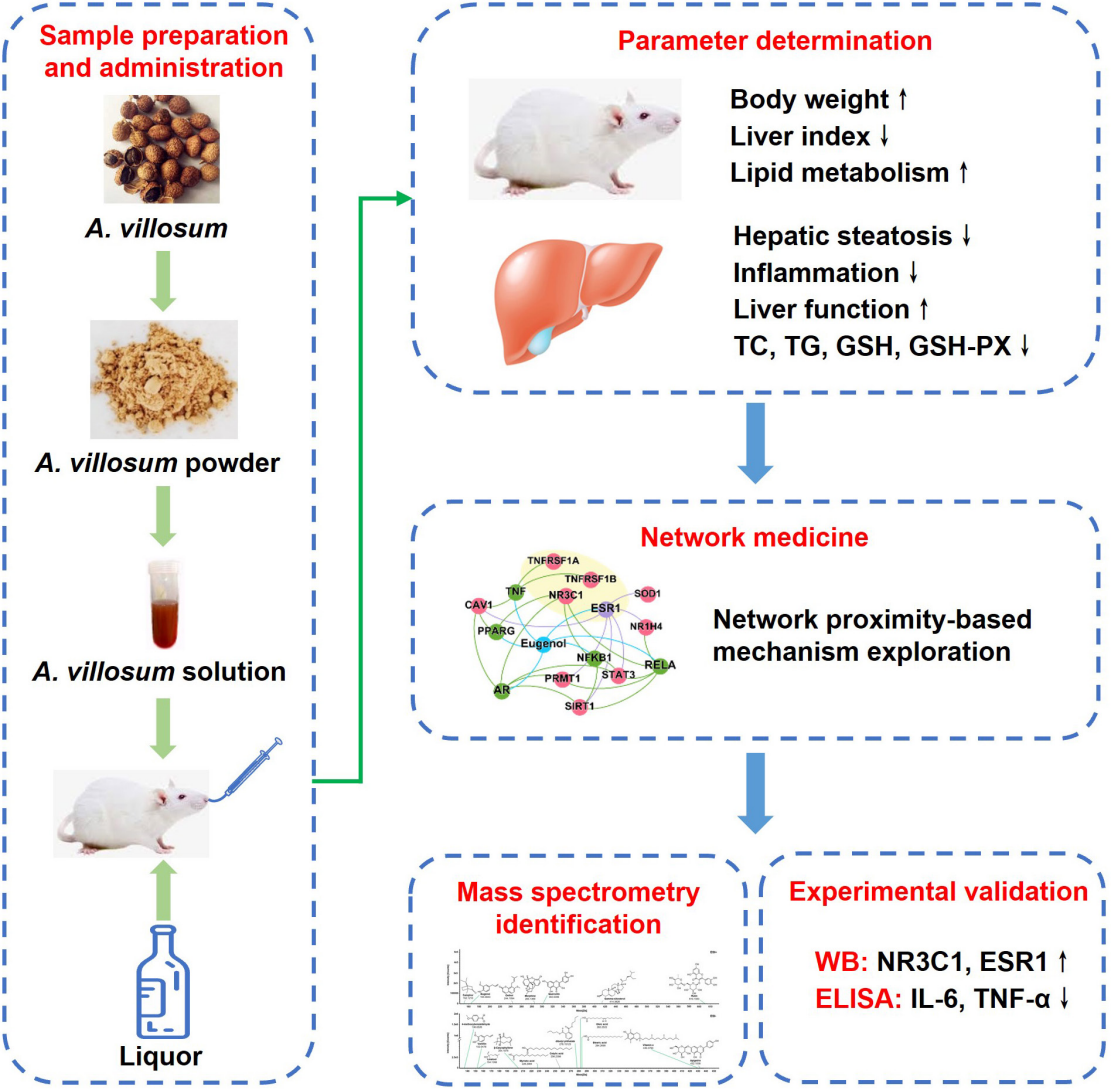
Diagram illustrating the network‐based active ingredient efficacy screening of *A. villosum* for ALD treatment.

Historically, *A*. *villosum* has been recognized in traditional medicinal systems, yet its pharmacological efficacy against ALD has largely been unexplored. This oversight is pronounced, given the potential of traditional remedies to serve as viable pharmacological interventions. Our research sought to address this issue by elucidating the hepatoprotective attributes of *A*. *villosum* using an ALD rat model. We pursued an integrated systems pharmacology strategy, as detailed in Figure 1. Our initial step centered on evaluating the pharmacodynamic influence of *A*. *villosum* on ALD within this model. Building on this, we applied network medicine concepts to identify the potentially active constituents of *A*. *villosum* and to chart their interconnected sub‐networks related to ALD. To ensure the rigor of our results, our study culminates in a validation step, in which the expression profiles of anticipated ALD‐correlated proteins were examined through both Western blotting and enzyme‐linked immunosorbent assay (ELISA) methods.

## 2 MATERIALS AND METHODS

### 2.1 Materials and reagents

The dried fruit of *A*. *villosum* was purchased from Yangchun City Spring Gold Agriculture Investment Co., Ltd. (Yangchun, China) and identified by Dr. Zhengming Qian (Dongguan HEC Cordyceps R&D Co., Ltd., Dongguan, China). The voucher specimen (ZMU‐20) was deposited in the research and development center. Assay kits for aspartate aminotransferase (AST), alanine aminotransferase (ALT), total bilirubin (TBIL), low‐density lipoprotein cholesterol (LDL‐C), high‐density lipoprotein cholesterol (HDL‐C), glutathione (GSH), glutathione peroxidase (GSH‐Px), total cholesterol (TC), and triglycerides (TG) were obtained from Nanjing Jiancheng Bioengineering Institute (Jiangsu, China). ELISA kits for rat tumor necrosis factor‐α (TNF‐α) and interleukin 6 (IL‐6) were purchased from Nanjing Senberga Biotechnology Co., Ltd. (Jiangsu, China). The bicinchoninic acid (BCA) protein assay kit, radioimmunoprecipitation assay (RIPA) lysis buffer, and enhanced chemiluminescence (ECL) solution were obtained from Beyotime (Shanghai, China). Primary antibody against estrogen receptor 1 (ESR1) was purchased from Santa Cruz Biotechnology (Santa Cruz, CA, USA), while anti‐nuclear receptor subfamily 3 group C member 1 (NR3C1) and glyceraldehyde‐3‐phosphate dehydrogenase (GAPDH) antibodies were obtained from Cell Signaling Technology (Beverly, MA, USA). All reagents used in this study were of analytical grade.

### 2.2 Preparation of *A. villosum* powder

*A. villosum* fruits were first cleaned, followed by the removal of the outer peel. After drying, the fruits were pulverized using a mechanical pulverizer. The resulting powder was sifted through a 100‐mesh sieve to ensure uniform granularity. The sieved powder was stored in a refrigerator at 4°C for further use.

### 2.3 Animals

Male Sprague–Dawley rats weighing 180–200 g were obtained from Huafukang Biotechnology Co., Ltd. (Beijing, China) (SCXK 2019‐0008). The rats were housed under controlled laboratory conditions (temperature: 23 ± 2°C, relative humidity: 60 ± 5%, and a 12:12‐h light/dark cycle) with ad libitum access to food and water. The study protocol was designed in strict compliance with ethical guidelines for animal experiments and was approved by the Animal Care and Ethics Committee of Zunyi Medical University (ZMU21‐2303‐061).

### 2.4 Animal experimental design

After a 7‐day acclimatization period, the rats were randomly allocated into five groups (*n* = 8): the control group (Con), vehicle group (Veh), silybin group (Sil, 40 mg/kg), *A*. *villosum* low‐dose group (20 mg/kg), and *A*. *villosum* high‐dose group (40 mg/kg). The selection of *A*. *villosum* dosages was based on the results of our preliminary studies. An appropriate amount of *A*. *villosum* powder was weighed and dissolved in distilled water to prepare suspensions with concentrations of 2 mg/mL and 4 mg/mL, respectively. Subsequently, the rats were treated by gavage administration in accordance with the body weights at a volume of 10 mL/kg. Except for the control and vehicle groups, the remaining groups received 10 mL/kg *A*. *villosum* suspension each morning. *A*. *villosum* is insoluble in aqueous solutions, and stirring was used to obtain a uniform suspension before each gavage. Two hours post‐administration, all groups, except for those in the control group, were given Chinese baijiu (Chinese white liquor, 56% by volume) named Red Star ErGuoTou (Beijing Red Star Co., Ltd., Beijing, China) at a dose of 10 mL/kg via gavage. This regimen was continued for 7 weeks, followed by a 12‐h fasting period after the final administration. Subsequently, all the rats were euthanized, and blood and liver tissue samples were collected for further analysis.

### 2.5 Histological analysis

Livers were harvested from each rat and weighed. Subsequently, the left lobe of the liver was excised, immediately fixed in 4% paraformaldehyde, dehydrated, embedded in paraffin, and sectioned into 4‐μm thick slices. These sections were then stained with hematoxylin and eosin (HE) to evaluate liver damage, as previously described (Huang et al., 2021). Hepatocellular steatosis was graded as follows: grade 0, no fat accumulation; grade 1, less than 33% of hepatic tissue showing steatosis; grade 2, 33%–66% of hepatic tissue showing steatosis; and grade 3, more than 66% of hepatic tissue showing steatosis.

### 2.6 Biochemistry analysis

Blood samples were collected by venipuncture and then centrifuged at 3000×*g* at 4°C for 10 min to obtain serum. The levels of AST, ALT, TBIL, LDL‐C, HDL‐C, TC, and TG were determined using the respective commercially available kits following the instructions of the manufacturers. For hepatic biochemical analysis, the right lobe of liver tissues (10 mg) was homogenized in 90 μL of saline at 65 Hz at 4°C for 2 min using a magnetic bead homogenizer (Jinxin Industrial Development Co., Ltd., Shanghai, China). The homogenate was then centrifuged at 3000×*g* at 4°C for 10 min, as previously described (Huang et al., 2023). The supernatant was collected, and the protein concentration was measured using a BCA protein assay kit. Subsequently, the levels of GSH, GSH‐Px, TC, TG, and the inflammatory cytokines TNF‐α and IL‐6 in the supernatant were determined using the appropriate assay kits.

### 2.7 Network medicine

#### 2.7.1 Constructing the human protein–protein interactome

The human interactome was established by drawing on 18 databases and relevant literature (Guney et al., 2016; Zhou et al., 2020). Experimentally validated, high‐quality human protein–protein interactions (PPIs) were compiled. Subsequently, the gene corresponding to the protein was mapped into Entrez IDs following the National Center for Biotechnology Information (NCBI) database, and a comprehensive human interactome was generated. The largest connected component (LCC), which encompassed 485,385 unique PPIs (edges) connecting 18,375 genes (nodes), was identified.

#### 2.7.2 Collection of ingredients of *A*. *villosum* and their targets

The traditional Chinese medicine systems pharmacology database (TCMSP) (Ru et al., 2014) and HERB database (Fang, Dong, et al., 2021) were used to identify the ingredients of *A*. *villosum*. The targets of these ingredients were identified using the DrugBank (Wishart et al., 2018), Comparative Toxicogenomics Database (CTD) (Davis et al., 2021), BindingDB (Gilson et al., 2016), and Ingenuity Pathway Analysis (IPA) database (Spasser, 1997). To ensure the quality of the data and the accuracy of the computational prediction, the finally included targets have an experimental basis.

#### 2.7.3 Collection of disease‐related genes

Initially, we collected causal genes associated with ALD defined by Medical Subject Headings (MeSH). The canonical name of the disease is associated with various synonyms, and we chose to search for “liver diseases, alcoholic,” “fatty liver, alcoholic,” “hepatitis, alcoholic,” and “liver cirrhosis, alcoholic” to ensure a comprehensive capture of related genes. To construct a high‐quality dataset, we corroborated the information using PubMed as a reference, specifically including only those genes directly associated with ALD, as evidenced by knockout mouse studies.

#### 2.7.4 Network proximity calculation

The network proximity calculation involved considering the distinct ingredients of *A*. *villosum* as individual drugs. Utilizing the methodology detailed in the relevant literature (Fang, Zhang, et al., 2021; Guney et al., 2016), we defined a set of drug targets (*T*) and a set of disease proteins (*S*) within a human interaction network. For a chosen target *t* within *T*, the shortest path length distance *d*(*s*, *t*) between *t* and all disease proteins *s* in *S* was calculated, identifying the minimum value, min *d*(*s*, *t*). This operation was repeated for all the other targets in *T*, resulting in ||*T*|| distances. To calculate the closest network distance (proximity), denoted as *d*_*c*_(*S*, *T*), we first computed the sum of these distances. This cumulative sum was then divided by the total count of drug targets. The mathematical formula representing this process is as follows:

$$d_{c}\left(S,T\right)=\frac{1}{∥T∥}\sum_{t\in T}\min_{s\in S}d\left(s,t\right)$$

To evaluate the significance of the network distance between a specific drug and disease, a reference distance distribution was constructed, mirroring the expected network topology between two randomly selected protein groups of matched size and degree to the original disease proteins and drug targets. This reference distance distribution was generated by calculating the distance between two randomly selected groups, and this operation was repeated 1000 times. We computed the relative closest network distance (*Z*‐score) using reference distribution, which was characterized by its mean distance, denoted as *μ*_d_(*S*, *T*), and standard deviation, denoted as *σ*_d_(*S*, *T*). The observed closest distance between the specified drug and disease, represented as *d*_c_(*S*, *T*), was standardized against this reference distribution to generate the *Z*‐score. A positive standardized score was obtained when the distance exceeded the mean distance, and a negative standardized score was obtained when the distance fell below the mean distance. The corresponding *p*‐value was computed based on permutation test results, with *Z*‐score <0 and *p* < .05 considered significant for the proximity of ingredient–ALD associations. To enhance the efficiency and accessibility of computational analyses in network proximity, we developed an online computational platform, which can be accessed at www.zmupredict.cn. This platform enables users to freely submit data for an array of calculations associated with network medicine. Currently, the platform supports three fundamental modules: network proximity calculation, sub‐network extraction, and combined drug analysis. Network proximity was translated into a *Z*‐score based on permutation tests, with a smaller *Z*‐score indicating a closer relative distance:

$$z\left(S,T\right)=\frac{d_{c}\left(S,T\right)-\mu_{d\left(S,T\right)}}{\sigma_{d\left(S,T\right)}}$$

#### 2.7.5 Network diagram construction

To construct a network involving drug targets and disease‐related proteins, we first mapped the ingredient target proteins and ALD proteins to the human interaction network. Subsequently, we extracted a sub‐network to generate the drug–disease subgraph utilizing the Python NetworkX package, as previously described (Airong Ren et al., 2024; Ren et al., 2023). The extracted node and edge lists were subsequently imported into Gephi software (version 0.9.2) to generate a comprehensive drug–disease diagram. Within these derived sub‐networks, we recognized the hub nodes as key ingredients of *A*. *villosum* in the interaction network for ALD.

### 2.8 Identification of predictive active ingredients in blood using mass spectrometry

Each serum sample (100 μL) was mixed with 300 μL of precooled methanol and acetonitrile (2:1, v/v). This mixture was then vortexed for 1 min to precipitate proteins. Subsequently, the samples were centrifuged at 4000×*g* for 20 min. The supernatant was characterized by a Waters Ultra‐High‐Performance Liquid Chromatography‐Quadrupole/Time‐of‐Flight–Tandem Mass Spectrometry (UPLC‐Q/TOF–MS/MS), as previously described (Ren et al., 2024; Ren et al., 2023). Chromatographic separation was performed on a Waters Acquity™ UPLC system using an ACQUITY UPLC BEH C18 column. Mass spectrometry analysis was performed on a Waters SYNAPT XS mass spectrometer in both positive and negative ion modes, with a mass range of 50–1200 Da. The data were analyzed using UNIFI software (version 1.8.2, Waters Corporation, USA) with the following specific parameters: chromatographic peak extraction time ranging from 0 to 20 min, with a response threshold greater than 500, and mass accuracy of ±10 ppm. Manual verification was employed to ensure the precise determination of the relative molecular masses of the compounds.

### 2.9 Western blot analysis

Liver samples (100 mg) were homogenized with 400 μL of RIPA lysis buffer, containing 1% phenylmethylsulfonyl fluoride (PMSF) and 1% phosphatase inhibitor cocktail and then homogenized at 4°C for 1 min. Following homogenization, the samples were centrifuged at 12,000 g at 4°C for 30 min. The supernatant was then isolated to determine the protein concentration using a BCA protein assay kit. As per our previously described method (Han et al., 2023; Zhang et al., 2022), the protein was loaded and separated through 15% sodium dodecyl sulfate‐polyacrylamide gel electrophoresis (SDS‐PAGE), and then transferred onto a polyvinylidene difluoride (PVDF) membrane. The membrane was blocked for 1 h at 25°C and then incubated overnight at 4°C with a primary antibody targeting either ESR1 or NR3C1. GAPDH served as the housekeeping protein. Following a TBST (Tris‐buffered saline with 0.1% Tween‐20) wash, the membranes were incubated with a secondary antibody for 1 h at room temperature. The membranes were subsequently treated with ECL solution and imaged using the ChemStudio system (Analytik Jena, Jena, Germany). Finally, the protein bands were semiquantitatively assessed using ImageJ software and normalized to β‐actin density.

### 2.10 Statistical analysis

The experimental data are presented as the mean ± 95% confidence interval (CI). The statistical examination of the data was performed using GraphPad Prism version 8.0 (GraphPad Software, San Diego, CA, USA). Differences among multiple groups were analyzed via one‐way analysis of variance (ANOVA), and *p* < .05 was considered to indicate statistical significance.

## 3 RESULTS

### 3.1 Effects of *A*. *villosum* on body weight and liver histology in rats with ALD

**FIGURE 2:**
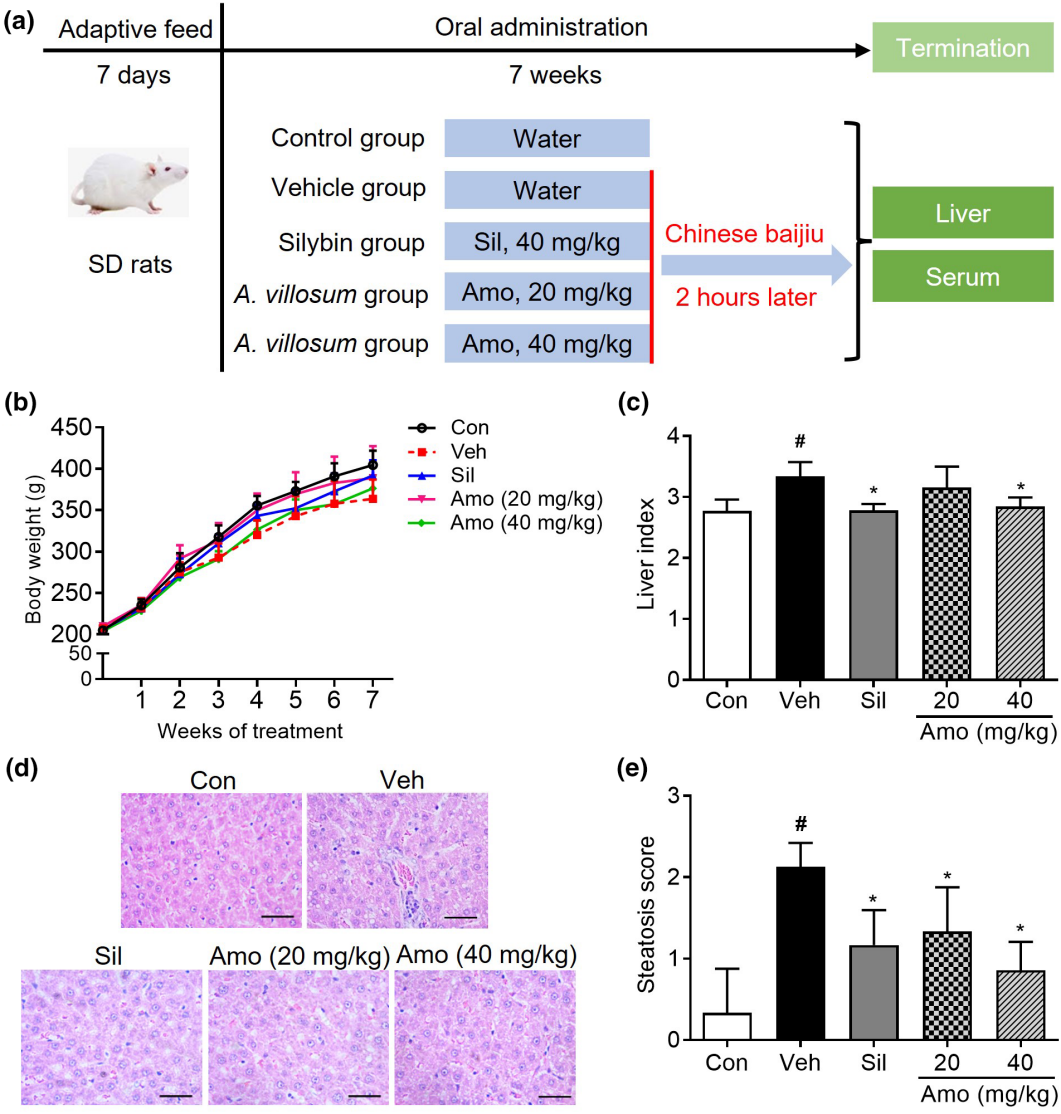
Effects of *A. villosum* on body weight and liver histology in an ALD rat model. (a) Flow diagram of the experimental groups. (b) Body weight and (c) hepatic index are presented. (d) Hematoxylin and eosin (HE)‐stained liver sections and (e) corresponding hepatic steatosis scores are shown. The scale bar indicates 100 μm. Data are presented as the mean ± 95% CI. ^#^ *p* < .05 compared with the Con group; *p < .05 compared with the Veh group. Amo, *A*. *villosum* group; Con, control group; Sil, silybin group; Veh, vehicle group.

Figure 2a outlines the different experimental groups used in the current study. During the experimental period of 1 to 7 weeks, there were no statistically significant differences in body weight variation between the control group (mean difference: 21.44 kg, 95% confidence interval: −63.31 to 106.20) and the groups administered either the vehicle or *A*. *villosum* (Figure 2b). Moreover, relative to that in the control group, the liver index in the vehicle group (mean difference: 0.56, 95% confidence interval: 0.24–0.90) increased markedly, whereas *A*. *villosum* administration (40 mg/kg) group (mean difference: 0.50, 95% confidence interval: 0.18–0.81) induced a significant reduction in liver index, as demonstrated in Figure 2c. Subsequently, HE staining was used to evaluate hepatic steatosis and inflammation. Histopathological examinations revealed severe hepatic steatosis and inflammation in the vehicle group, conditions that were effectively mitigated following the administration of *A*. *villosum* (Figure 2d). Steatosis scores were elevated in the vehicle group relative to those in the control group, while *A*. *villosum* treatment (20 mg/kg) group (mean difference: 0.79, 95% confidence interval: 0.18–1.40) and *A*. *villosum* treatment (40 mg/kg) group (mean difference: 1.27, 95% confidence interval: 0.69–1.86) led to a significant reduction in steatosis scores in rats suffering from alcohol diet‐induced ALD (Figure 2e).

### 3.2 Effects of *A*. *villosum* on the serum levels of TC, LDL‐C, HDL‐C, AST, ALT, and TBIL in ALD rats

**FIGURE 3:**
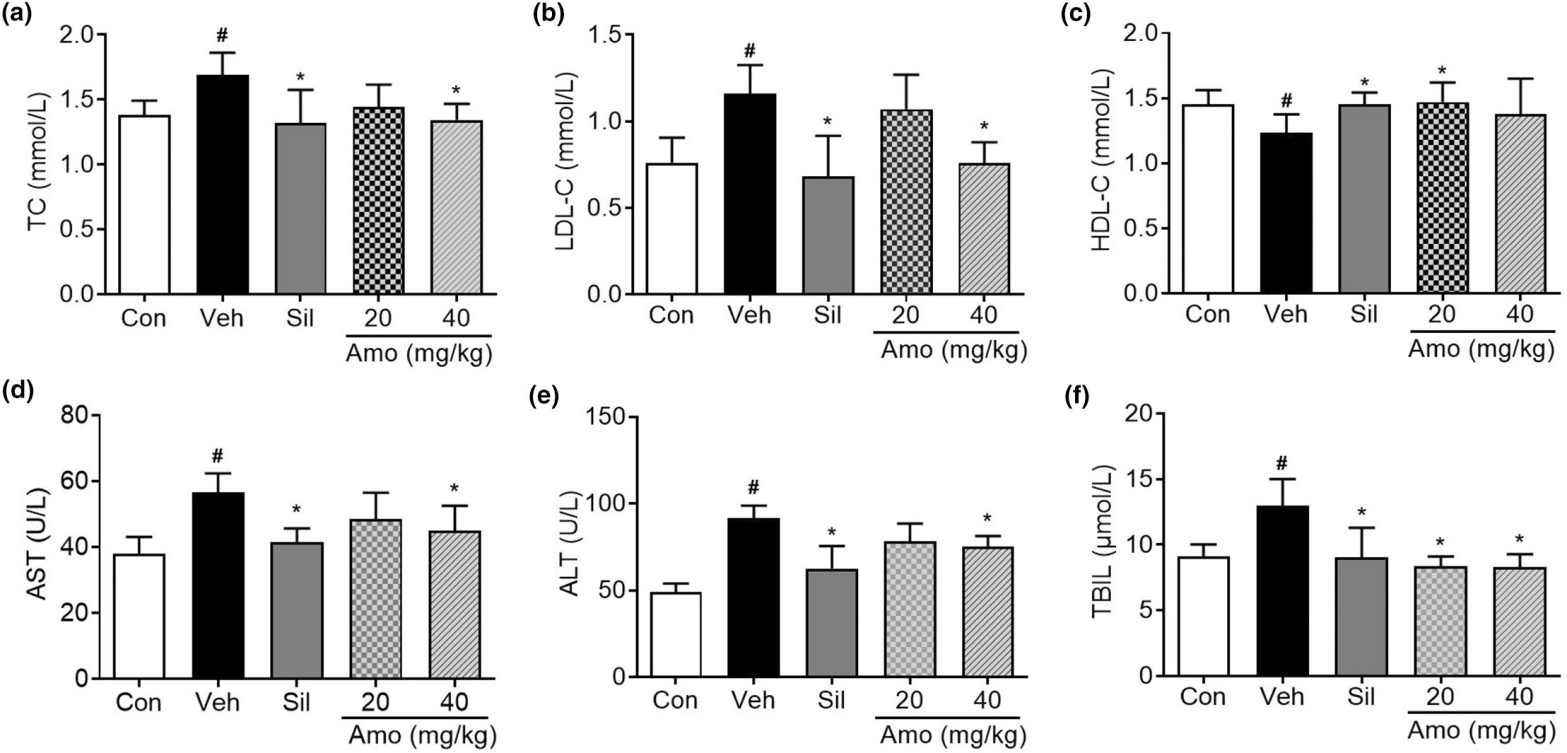
Effects of *A. villosum* on serum lipid metabolism and liver function in an ALD rat model. The serum levels of (a) TC, (b) LDL‐C, and (c) HDL‐C and liver function indicators, including (d) AST, (e) ALT, and (f) TBIL, are shown. Data are expressed as the mean ± 95% CI. ^#^ *p* < .05 compared with the Con group; *p < .05 compared with the Veh group. Amo, *A*. *villosum* group; Con, control group; Sil, silybin group; Veh, vehicle group.

Histopathological examination indicated that *A*. *villosum* effectively mitigated alcohol diet‐induced hepatic steatosis and inflammation. Moreover, *A*. *villosum* led to a decrease in the serum TC and LDL‐C levels and an increase in the serum HDL‐C level, suggesting its ability to normalize blood lipid metabolism (Figure 3a–c). Concurrently, the serum AST, ALT, and TBIL levels in the vehicle group were significantly increased than those in the control group. In contrast, treatment with *A*. *villosum* (40 mg/kg) led to significant reductions in AST, ALT, and TBIL levels (Figure 3d–f). These findings are consistent with the pathological observations and further strengthen the argument for the protective efficacy of *A*. *villosum* against ALD.

### 3.3 Effects of *A*. *villosum* on liver homogenate TC, TG, GSH‐Px, and GSH in rats with ALD

**FIGURE 4:**
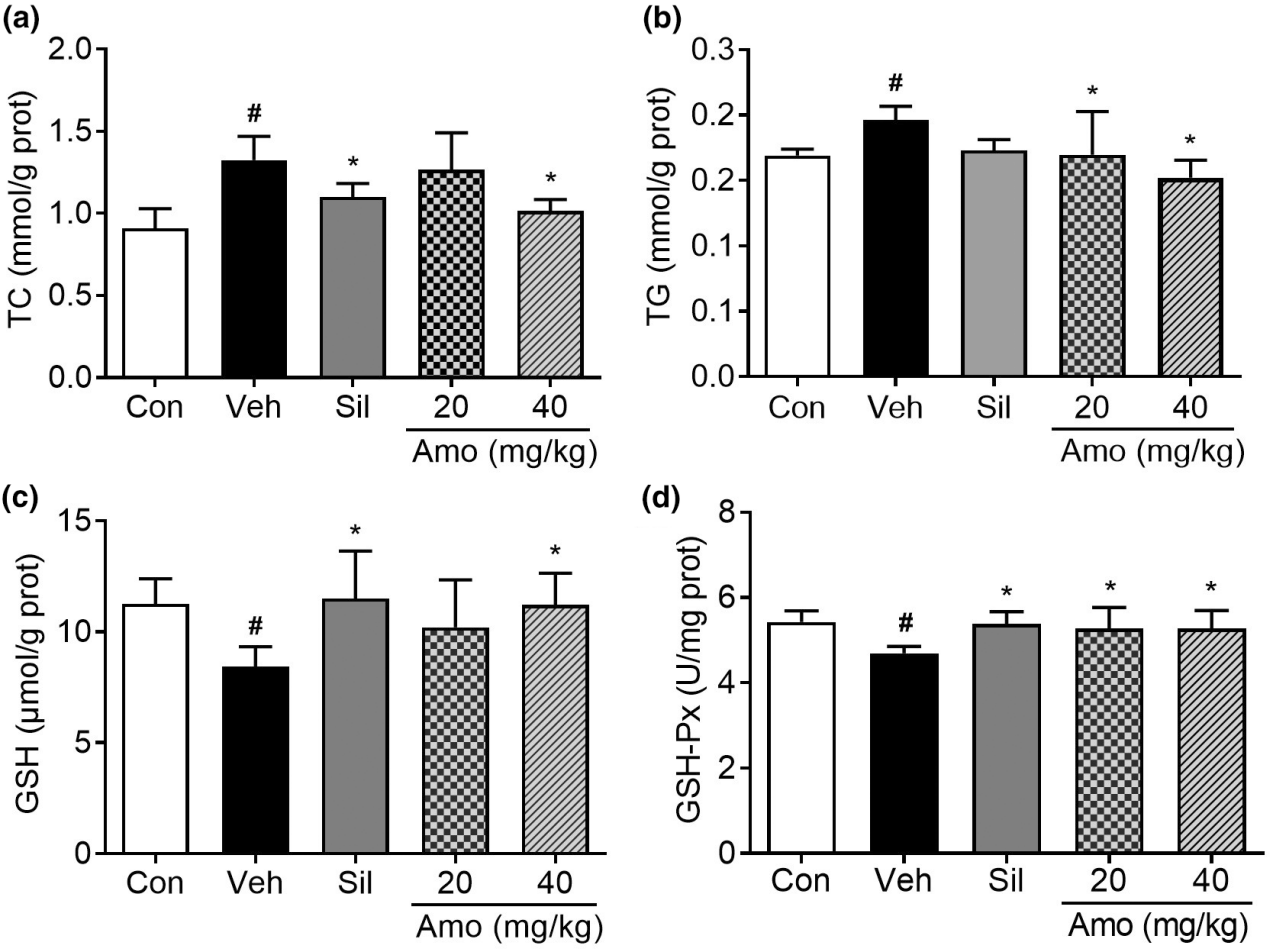
Effects of A. villosum on lipid metabolism and oxidative stress in the liver homogenates of ALD rats. The levels of TC (a), TG (b), GSH (c), and GSH‐Px (d) are shown. Data are represented as the mean ± 95% CI. ^#^ *p* < .05 compared with the Con group; *p < .05 compared with the Veh group. Amo, A. villosum group; Con, control group; Sil, silybin group; Veh, vehicle group.

As shown in Figure 4a,b, *A*. *villosum* administration yielded reductions in hepatic TC and TG levels relative to the vehicle group. GSH and GSH‐Px play crucial roles as antioxidants in the liver. Additionally, treatment with *A*. *villosum* counteracted the ethanol‐induced increase in hepatic GSH‐Px and GSH levels, with the 40 mg/kg dose demonstrating a significant effect (Figure 4c,d). These results suggest that *A*. *villosum* exhibits hepatoprotective properties through the regulation of lipid metabolism and the alleviation of oxidative stress, thus providing a foundation for potential interventive applications of *A*. *villosum* in preventing and treating ALD.

### 3.4 *A. villosum* ingredient–target network analysis

**FIGURE 5:**
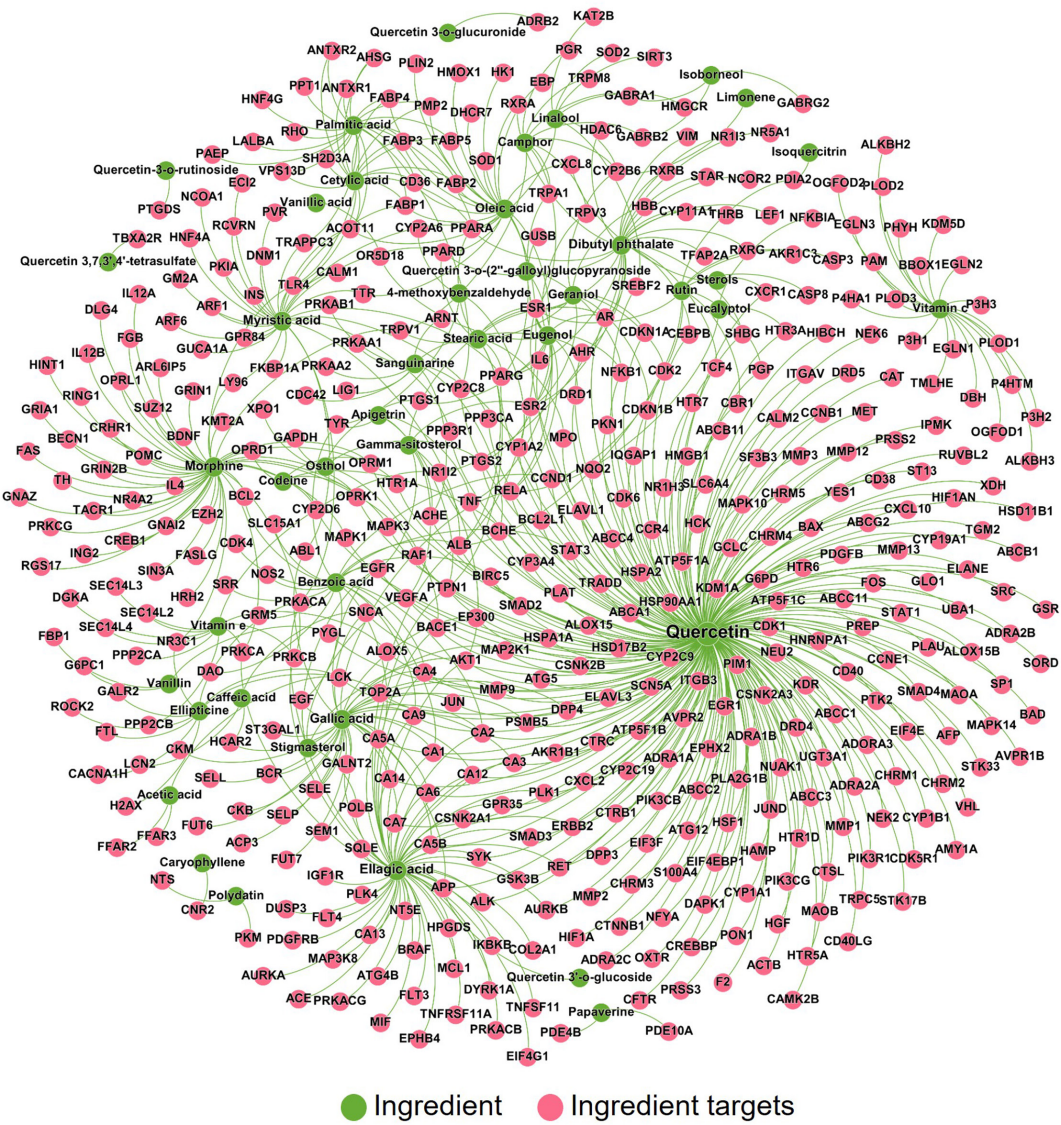
Ingredient–target network of key *A. villosum* ingredients. The network comprises 2675 interactions, connecting 43 ingredients (highlighted in green) and 462 protein targets (highlighted in red). The size of the node and the font size of the label are proportional to the degree of connectivity.

A comprehensive network analysis elucidated the complex interactional relationship between the ingredients of *A*. *villosum* and their corresponding molecular targets. In total, 677 ingredient–target pairs were included in this study (Table S1). As represented in Figure 5, the constructed network visualizes 675 interactions, connecting 43 ingredients (depicted in green) with 462 distinct protein targets (represented in red). Examination of this intricate network structure enables in‐depth exploration of the complex interconnections between the bioactive ingredients of *A*. *villosum* and their associated protein targets.

### 3.5 Network proximity‐based prediction of the active ingredients of *A*. *villosum* for ALD

In our subsequent analysis, we investigated the network proximity between *A*. *villosum* ingredient targets and ALD proteins within the human interactome. From the literature, we identified 34 genes that are known to be associated with ALD (Table S2). All network proximity scores are provided in Table S3. Table 1 presents the results of a network medicine‐based exploration, which revealed plausible interactive relationships between an array of *A*. *villosum* ingredients and ALD. Guided by the principle of prioritizing elements with a *Z*‐score <0 and *p* < .05, the study ranks active ingredients according to ascending *Z*‐scores. In total, 18 potentially active ingredients of *A*. *villosum* were predicted for ALD treatment. For instance, caryophyllene and stearic acid, which are connected by a distinct ingredient target, exhibited the most pronounced negative *Z*‐scores (−5.58 and − 4.30, respectively), thereby indicating their significant potential for intervention in ALD. Similarly, notable negative *Z*‐scores associated with eugenol (*Z*‐score = −3.78) and dibutyl phthalate (*Z*‐score = −3.37) underscore their potential interventive value. These observations suggest that multiple ingredients of *A*. *villosum* may confer beneficial effects in treating ALD, thereby warranting further research.

**TABLE 1:** Predicted associations between *A. villosum* ingredients and ALD.

Rank	Ingredient	*Z*‐score	*p*‐value	References
1	β‐Caryophyllene	−5.58	<.001	(Varga et al., 2018)
2	Stearic acid	−4.30	<.001	(Nie et al., 2022)
3	Eugenol	−3.78	<.001	(Anbu & Anuradha, 2012)
4	Dibutyl phthalate	−3.37	.005	–
5	Myristic acid	−3.26	.009	–
6	Gamma‐sitosterol	−3.19	.000	–
7	Morphine	−2.87	.013	–
8	Linalool	−2.81	.005	–
9	Oleic acid	−2.74	.012	–
10	Quercetin	−2.67	.011	(S. Lee et al., 2019)
11	Vanillin	−2.49	.005	(Haseba et al., 2008)
12	Osthol	−2.39	.002	(Sun et al., 2010)
13	Rutin	−2.28	.009	(C. C. Lee et al., 2013)
14	Cetylic acid	−1.98	.019	(You et al., 2005)
15	4‐methoxybenzaldehyde	−1.90	.010	–
16	Camphor	−1.66	.026	–
17	Vitamin E	−1.35	.039	(Kaur et al., 2010)
18	Apigetrin	−1.06	.032	–

*Note*: Ingredients were systematically ordered based on the network proximity (indicated by the *Z*‐score) of their associated targets to the genes linked with ALD, adopting the stringent criterion of *Z*‐score <0 and *p* < .05. The indicated references drawn from meticulously reviewed literature are provided. The symbol “–” is used to denote instances where specific information is not provided or reported. 4‐MET: 4‐methoxybenzaldehyde.

### 3.6 Identification of the active ingredients in rat serum using UPLC‐Q/TOF–MS/MS

**FIGURE 6:**
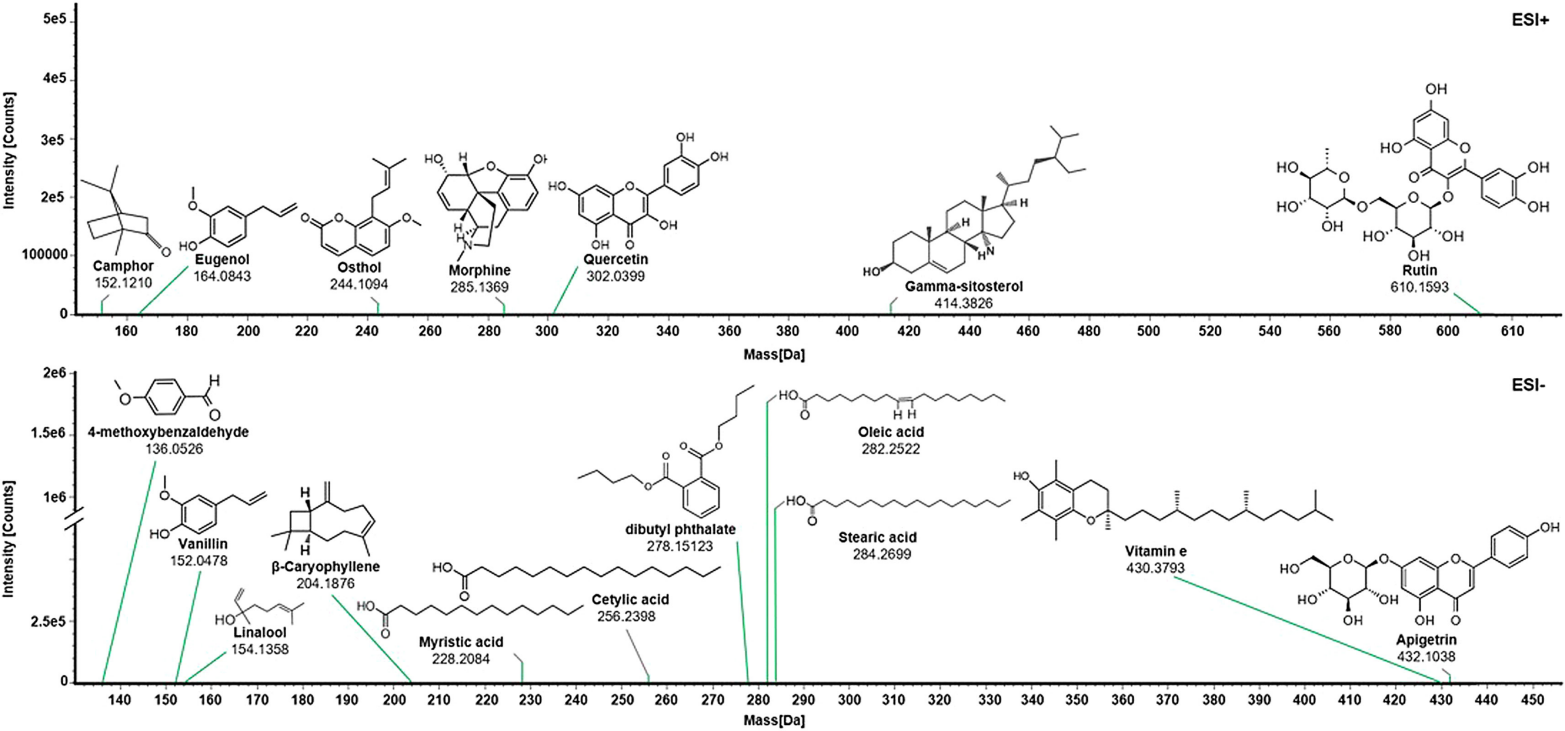
Identification of 18 prototype network medicine‐based predicted active ingredients in rat serum by UPLC‐Q/TOF–MS/MS. Mass spectra of each peak were obtained and used for compound identification.

Following the network proximity‐based prediction of active ingredients, we identified these components in rat serum using a high‐resolution, sensitive UPLC‐Q/TOF–MS/MS system. Figure 6 illustrates the chromatographic peaks of the 18 predicted active ingredients detected within the rat serum samples. This identification not only confirms their systemic absorption but also suggests their potential role in the observed therapeutic effects in the rat model.

### 3.7 Potential mechanism‐of‐action of *A*. *villosum* by network medicine inference and experimental validation

**FIGURE 7:**
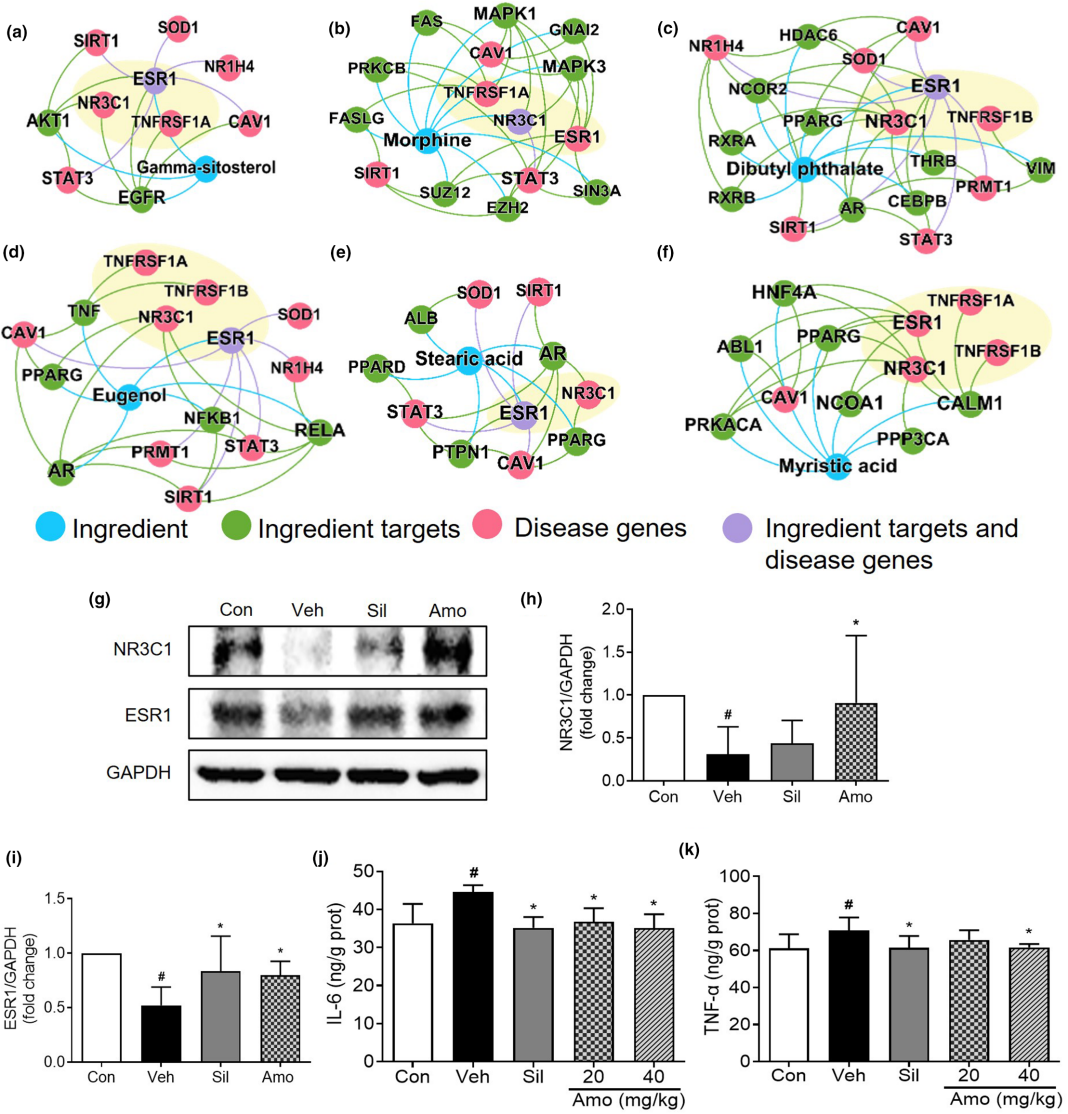
Potential proteins predicted via network‐based rational screening of specific chemical ingredients within *A. villosum*. The sub‐network of gamma‐sitosterol (a); morphine (b); dibutyl phthalate (c); eugenol (d); stearic acid (e); and myristic acid (f). The protein expression levels of NR3C1 and ESR1 in the rat liver were detected by Western blot analysis (g). Relative quantitative analysis of NR3C1 (h) and ESR1 (i) was performed. The protein expression levels of IL‐6 (j) and TNF‐α (k) in the rat liver were assessed via ELISA. The values are expressed as the mean ± 95% CI. ^#^ *p* < .05 compared with the Con group; *p < .05 compared with the Veh group. Amo, *A*. *villosum* group; Con, control group; Sil, silybin group; Veh, vehicle group.

Next, we focused on six primary ingredients, namely gamma‐sitosterol, morphine, dibutyl phthalate, eugenol, stearic acid, and myristic acid. These constituents were chosen due to their interaction with more than two targets and their display of the lowest *Z*‐scores. The intricate ingredient–target and disease–target sub‐networks associated with these ingredients are illustrated in Figure 7a–f. The intersections between the ingredient–target and disease–target categories are highlighted in purple, accentuating their potential relevance.

Further analysis through Western blot verification results demonstrated that the expression levels of NR3C1 and ESR1 were significantly reduced (*p* < .05) in the vehicle group than in the control group. In contrast, compared with those in the vehicle group, the expression levels of NR3C1 and ESR1 in the *A*. *villosum* group (40 mg/kg) were significantly increased (*p* < .05, Figure 7g–i). Concurrent with these findings, ELISA revealed that the hepatic levels of IL‐6 and TNF‐α were dramatically increased in the vehicle group than in the control group, but these levels were substantially reduced following *A*. *villosum* administration (Figure 7j,k). Within each sub‐network diagram, proteins were confirmed through experimental validation and are distinctly highlighted in light yellow. These findings suggest that the hepatoprotective effects of *A*. *villosum* on ALD could be mediated through the regulation of NR3C1, ESR1, IL‐6, and TNF‐α.

## 4 DISCUSSION

This study provides compelling evidence for the hepatoprotective effects of *A*. *villosum* in rats suffering from ALD, which is characterized by the ability of the traditional medicinal herb to mitigate liver steatosis, inflammation, and alterations in lipid metabolism, as well as oxidative stress, ultimately promoting liver function. Network medicine methodologies were instrumental in elucidating the potential key ingredients of *A*. *villosum* and the proteins involved in liver protection, offering intriguing insights and potential trajectories for its hepatoprotective attributes. Western blotting and ELISA were employed to validate these potential proteins and ascertain the underlying protective mechanisms, adding considerable value to the understanding of the pharmacological effects of *A*. *villosum* and its potential interventional applications for liver protection. Our study possesses three important implications. Initially, we examined the pharmacological impacts of *A*. *villosum* on alcohol‐induced chronic liver injury in rats, thus providing evidence that bolsters its clinical use as a medicinal herb. Second, we anticipated potentially active ingredients for ALD through the strategic application of network medicine proximity computations, a step that could streamline the process of novel drug development for this ailment. Finally, our findings pave the way for the establishment of a quality marker (Q‐marker) for *A*. *villosum*, providing a promising foundation for future research endeavors in this domain.

Embarking on our study, we first examined the hepatic index, an indicator commonly employed for liver edema evaluation, which is determined by dividing the liver weight to body weight (Zhang et al., 2019). In our investigation, we noted a significant elevation in the hepatic index in the vehicle group compared to that in the control group, suggesting the presence of substantial liver edema. In contrast, treatment with *A*. *villosum*, administered at a dosage of 40 mg/kg, resulted in a marked reduction in the hepatic index relative to that in the vehicle group, thereby underscoring the potential of the herb for curtailing liver edema. We further ventured into the biochemical realm of lipid metabolism, where disruptions in lipid metabolism constitute a predominant characteristic of ALD, attributable to the influence of alcohol on hepatic lipid metabolism and transport (Sozio et al., 2010). LDL‐C and HDL‐C, crucial apolipoproteins implicated in lipid transportation, have been demonstrated to play significant roles in this process. The present study provided evidence in line with the findings of Kang et al. (Mahdi et al., 2023), who identified similar elevations in the serum levels of total TC, TG, and LDL‐C, as well as a decrease in HDL‐C levels, after alcohol intake. Notably, the application of *A*. *villosum* in our study demonstrated pronounced amelioration of these lipid metabolic perturbations. Drawing further insights from our findings, *A*. *villosum* has shown promise in mitigating the effects of ALD in rats, as demonstrated by a significant reduction in the hepatic index and notable regulation of lipid metabolism, suggesting its potential utility as a hepatoprotective agent.

Assessment of liver function primarily hinges on the evaluation of the serum levels of AST, ALT, and TBIL, which provide insights into the severity of hepatic steatosis and liver injury (Sorbi et al., 1999). A surge in the membrane permeability of liver cells can lead to the leakage of AST and ALT into the bloodstream, thereby increasing their levels. The serum TBIL, which mainly originates from hemoglobin released during erythrocyte lysis, is closely regulated by hepatocytes and is essential for bilirubin uptake, conjugation, and excretion (Zhong et al., 2021). Our study demonstrated a significant reduction in the serum AST, ALT, and TBIL levels following *A*. *villosum* treatment, highlighting its potential interventional impact on alcohol‐induced liver injury.

Besides the disruptions in lipid metabolism, alcohol‐induced liver injury is also influenced by other key molecular mechanisms, particularly oxidative stress. This oxidative imbalance, characterized by a disproportionate ratio of oxidants to antioxidants, is a pivotal factor in lipid peroxidation (Yurt & Celik, 2011). Previous studies have corroborated the link between alcohol‐induced liver damage and oxidative stress markers, such as increased lipid peroxidation, escalated production of free radicals, and diminished liver antioxidant capabilities (Cui et al., 2014; Zeng et al., 2012). Under normal physiological conditions, intracellular antioxidants, such as GSH and GSH‐Px, effectively neutralize free radicals, maintaining oxidant equilibrium. However, alcohol consumption can disrupt this equilibrium, leading to excessive antioxidant depletion and consequent liver damage (Li et al., 2015). Our study revealed that *A*. *villosum* can increase GSH and GSH‐Px levels, thereby restoring oxidative equilibrium and suggesting a potential therapeutic approach for alcoholic liver injury. These findings are consistent with those of Gao et al., whose study demonstrated that a notable compound from *A*. *villosum* markedly increased GSH levels in BV‐2 cells by activating the nuclear factor erythroid‐2 related factor 2/heme oxygenase 1 (NRF2/HO‐1) pathways, indicating its efficacy in oxidative regulation (Gao et al., 2023).

Next, we applied a network‐based ingredient screening model and identified 18 key ingredients based on their Z‐scores. Among these, β‐caryophyllene was observed to mitigate both chronic and binge alcohol‐induced liver damage and inflammation. This effect is accomplished by tempering the M1 pro‐inflammatory phenotype switch in Kupffer cells and curtailing the expression of vascular adhesion molecules (Varga et al., 2018). Equally noteworthy is stearic acid, which was reported to prevent or alleviate ALD via the modulation of gut microbiota and bolstering of the gut barrier (Nie et al., 2022). Similarly, eugenol effectively reduces oxidative stress and enhances both enzymatic and nonenzymatic antioxidant levels in the plasma of alcohol‐fed rats (Anbu & Anuradha, 2012). Along with its glycoside derivatives, quercetin contributes to combating ethanol‐induced hepatotoxicity by reducing hepatic transaminase activity and minimizing inflammatory responses in HepG2 cells (Lee et al., 2019). Furthermore, the administration of vanillin to ethanol‐intoxicated mice has been shown to inhibit ethanol metabolism, thereby resulting in reduced blood acetaldehyde concentrations (Haseba et al., 2008). Osthole, a potent ingredient isolated from osthol, has demonstrated promising results in improving lipid accumulation and reducing lipid levels in the serum and liver tissues of mice and rats afflicted with alcoholic fatty liver disease (Sun et al., 2010). In another notable instance, rutin exhibits hepatoprotective effects by inhibiting the elevation of serum AST, ALT, and alkaline phosphatase levels in animals subjected to ethanol and CCl_4_ induction (Lee et al., 2013). Cetylic acids, when administered to ethanol‐treated rats and 3T3‐L1 adipocytes, increase adiponectin secretion and enhance the activity of the mouse adiponectin promoter, thus exerting a protective influence against alcoholic fatty liver disease (You et al., 2005). Notably, previous investigations demonstrated the potential of vitamin E to alleviate the detrimental effects of alcohol on the livers of mice. This mitigation is evidenced by the restoration of redox equilibrium, a decrease in apoptosis rates, and a reduction in oxidative stress markers, thereby underscoring the therapeutic potential of vitamin E in alcohol‐induced liver damage (Kaur et al., 2010). Collectively, these findings suggest that these compounds could hold promise for intervention in ALD, meriting further exploration.

Considering the predicted sub‐network derived from network medicine, we experimentally verified the effects of *A*. *villosum* on ESR1, NR3C1, TNF‐α, and IL‐6 through Western blot and ELISA methods. The experimental results revealed that ESR1 and NR3C1 protein expression levels were elevated in the *A*. *villosum* group compared to those in the vehicle group, whereas TNF‐α and IL‐6 levels were significantly reduced. ESR1, a potent transcription factor, is recognized for its instrumental role in orchestrating hepatic glucose and lipid homeostasis, as well as in regulating hepatic cholesterol output. Decreased ESR1 expression level can lead to conditions such as dyslipidemia and steatosis (Jia et al., 2015). This viewpoint gains further support from a noteworthy investigation by Khristi et al. (Khristi et al., 2019), which highlights how ESR1 disruption leads to altered expression of genes governing hepatic lipid and carbohydrate metabolism in male rats. Furthermore, in an experimental context in which mice were fed a high‐fat–high‐sugar diet, liver‐specific knockout of NR3C1, also known as glucocorticoid receptor, was shown to exacerbate steatohepatitis (Lu et al., 2022). In contrast, our findings indicate that the application of *A*. *villosum* reversed the decrease in NR3C1 expression in a rat model of ALD. Moreover, the well‐documented inflammatory mediators such as TNF‐α and IL‐6, which are primarily secreted by Kupffer cells and peripheral blood mononuclear cells (PBMCs), are known as key contributors to alcoholic liver injury (Chen et al., 2014; Zhou et al., 2018). By integrating our research findings with the diverse body of scientific literature, we contend that the hepatoprotective benefits of *A*. *villosum* in ALD could be linked to its ability to influence the protein expression of ESR1, NR3C1, TNF‐α, and IL‐6.

## 5 CONCLUSIONS

In conclusion, our investigation compellingly demonstrated that *A*. *villosum* imparts considerable protection against ALD. This protective effect manifested chiefly through a decrease in the liver index in rats, favorable regulation of blood lipid metabolism, marked improvement in liver function, increase in antioxidant capacity, and alleviation of both hepatic steatosis and inflammation. Utilizing the power of network medicine, we successfully predicted potential intervention targets of *A*. *villosum* for combating ALD. This discovery provides us with fresh insights and possibilities for developing new intervention strategies. Ultimately, our study not only paves the way for the clinical application of *A*. *villosum* but also stimulates renewed interest and innovative thought in the prevention and treatment of ALD by using TCM.

## AUTHOR CONTRIBUTIONS

**Jing Wei:** Data curation (equal); investigation (lead); methodology (lead); project administration (equal); writing – original draft (equal). **Sihua Wang:** Methodology (equal); writing – review and editing (equal). **Junze Huang:** Validation (equal); visualization (equal); writing – review and editing (equal). **Xinhua Zhou:** Conceptualization (equal); formal analysis (lead); writing – review and editing (equal). **Zhengming Qian:** Conceptualization (equal); validation (equal); writing – review and editing (equal). **Tingbiao Wu:** Formal analysis (equal); investigation (equal); methodology (equal). **Qing Fan:** Data curation (lead); funding acquisition (equal); methodology (equal). **Yongyin Liang:** Data curation (equal); visualization (equal). **Guozhen Cui:** Conceptualization (equal); funding acquisition (lead); investigation (lead); project administration (lead); supervision (lead); writing – review and editing (lead).

## CONFLICT OF INTEREST STATEMENT

The authors declare that there are no conflicts of interest.

## ETHICS STATEMENT

The study protocol was designed in strict compliance with the guidelines of the national standards outlined in “Laboratory Animal Requirements of Environment and Housing Facilities” (GB 14925–2010), and animal experiments were approved by the Animal Ethics Committee of Zunyi Medical University (Approval No. ZMU21‐2303‐061).

## Supporting information


Table S1.

Table S2.

Table S3.

## Data Availability

The data that support the findings of this study are available from the corresponding author upon reasonable request.
